# The Hookup: Collaborative Evaluation of a Youth Sexual Health Program Using Text Messaging Technology

**DOI:** 10.2196/mhealth.3583

**Published:** 2014-11-03

**Authors:** Bhupendra Sheoran, Rebecca A Braun, Jenna Patrice Gaarde, Deborah K Levine

**Affiliations:** ^1^Internet Sexuality Information Services, Inc (dba YTH)Oakland, CAUnited States; ^2^California Family Health CouncilBerkeley, CAUnited States

**Keywords:** sexual health, STDs, HIV, mobile phone, youth, SMS, text messaging, program evaluation

## Abstract

**Background:**

The Hookup is a collaborative project reaching young people in California with valuable sexual and reproductive health information and linkage to local resources. Due to limited access to subscriber contact information, it has been a challenge to evaluate the program.

**Objective:**

The aims of this study were to determine the feasibility of using text messaging (short message service, SMS) as an evaluation tool for an educational text message-based program and to evaluate the program itself.

**Methods:**

All subscribers of The Hookup were sent four survey questions via SMS about age, gender, location, referral source and behavior change. An incentive was offered for completing the survey and an opt-out option was provided in the initial message.

**Results:**

All existing subscribers of The Hookup (N=2477) received a request to complete the survey using the SMS application on their mobile phones. A total of 832 (33.6%) subscribers responded to the initial question and 481 (20%) answered all four questions. Of the responses, 85% were received in the first two hours of the initial request. Respondents who answered the question about behavior change, 90% reported having made some positive change since subscribing to Hookup, including getting tested for STDs and HIV.

**Conclusions:**

The survey methodology initiated a high response rate from The Hookup subscribers. The survey was able to provide data about subscribers in a short time period at minimal cost. The results show potential for using mobile SMS applications to evaluate SMS campaigns. The findings also support using SMS to provide young people with sexual health prevention messaging and linkage to health services.

## Introduction

Mobile phone use has exploded in the United States (US) and across the world in the last decade, providing a cheap, easy and fast way to communicate with others. Five billion text messages (short message service, SMS) are sent daily in North America [1]. The volume of texting among teens has risen from 50 texts per day in 2009 to 60 texts in 2011 for the median teen texter. Of all teens, 63% say they exchange text messages with people everyday. This far surpasses the frequency with which they use other forms of daily communication [2,3].

Preliminary data has shown that SMS can be used successfully to increase knowledge around reproductive health issues [4,5], as well as to promote short-term behavior change for sexual and reproductive health [6-8]. Behavior changes included intent to use condoms, increased access to reproductive and sexual health services, including sexually transmitted disease (STD) and HIV testing, and intent to have fewer sex partners.

A study in South Africa used a randomized controlled study design to test whether SMS messages could motivate people to test for HIV [9]. Participant interactions were conducted via SMS and multimedia message service (MMS). The study found that 10 motivational-style SMS/MMS messages encouraged a statistically significant amount of people to test for HIV as compared to the control group. Another randomized control study in Australia, which used SMS as a health promotion tool, found a significant increase in STD knowledge and STD testing in the intervention group as compared to the control [10].

There exists a small yet limited database of using SMS for sexual health research in the context of randomized control trials [11]. Such information has typically been collected through surveys and interviews conducted face to face, by telephone, or online. These methods have the disadvantage of being costly and time consuming. According to a recent meta analysis, half of all online surveys received less than a 26% response rate, with the majority of responses (96.5%) taking two weeks to arrive [12,13]. Additionally, response rates to telephone surveys have continuously declined over the past decade [14, 15].

As SMS is the most commonly used mobile app worldwide, extremely fast and highly reliable [16], known to be opened and read in short time after delivery [17], this study aimed to discover if SMS could be a feasible tool to use to collect user data, particularly from a program concerning sensitive content around sexual and reproductive health for teens. 

Designed and implemented as a partnership between California Family Health Council (CFHC) and youth+tech+health (YTH) in April 2009, The Hookup is a California statewide sexual health text messaging service for adolescents between the ages of 13 and 24. The goals of the program are to connect users with relevant and accurate sexual and reproductive health information, and serve as a convenient, confidential resource for accessing local clinics that offer free or low-cost sexual and reproductive health services.

By sending the word “Hookup” as a text message to 61,827, young people subscribe for weekly sexual health tips. Subscribers can also enter the word “Clinic” plus their zip code to obtain local clinic referrals for youth-friendly, free and low cost STD/HIV testing, and reproductive health services. Subscribers are also linked to Teensource, a youth-focused sexual health website, for further information. All content for the messages is developed through inputs from the youth community and reviewed by internal medical, sexual health, and adolescent experts. Promotion of the Hookup was conducted through outreach in school-based health centers, other community-based partnerships using print materials, social media, and through the Teensource website. To increase sign-ups, we distributed branded youth-selected collateral such as lip balm, palm cards, condom tins, and keychains. While the program staff has access to subscribers’ cell phone numbers, this access is for the sole purpose of sending The Hookup messages. This has posed a challenge in evaluating the program, and led to the design of an SMS-based survey tool.

## Methods

### Aims

The aims of this study were: (1) to assess the feasibility of using SMS as an evaluation tool for a text message-based sexual health program for California youth, and; (2) to evaluate the effectiveness of the program itself.

### Feasibility of SMS Evaluation

At the time of the survey in January 2011, The Hookup had 2477 subscribers receiving weekly messages. The evaluation team, comprised of members from the community, CFHC and YTH, identified and developed the survey questions (Textbox 1) adhering to the 160 character limitation of a standard text message in the United States. Additional elements included: specific format of responses “Text ___”; examples to avoid confusion “Like ___”; requirement for respondent to be concise.

The evaluation format included an introductory text message with an opt-out option, four survey question messages and a concluding message. The first three questions had limited response options, while the fourth question was open-ended. The text evaluation was conducted on the day and at the same time that the usual weekly messages were released. In order to increase response rates, the team offered an incentive in the form of a draw for US $50 Target gift cards for 10 subscribers who completed the survey.

All responses were exported from the SMS platform to excel to conduct the analysis and included phone number, date/time of response, and body of response. The evaluation team additionally tracked response time for completing the survey and compared it to traditional survey data collection methods.

### Evaluation of The Hookup

The three main questions that the evaluation of the program looked to answer were: (1) are we reaching target population (California youth)?; (2) are our promotion efforts successful?; and (3) are our messages effective?

Throughout the program, YTH collected aggregate process data during the first year of operations to track overall program usage. This included the number of new subscribers, opt-outs, and clinic searches. In order to evaluate the program by answering the evaluation questions, in 2011 YTH and CFHC designed and conducted the following survey to understand the demographics of the population, how they heard about the program, and potential impact in terms of behavior change. For the program evaluation, The Hookup subscribers were asked to answer four questions about their age and gender, location, referral source, and behavior change efforts using an SMS survey via their mobile phones (Textbox 1).

**Survey questions.**

Hookup: No tip this week. Your turn to help the Hookup! 4 questions coming your way. Answer all and you can win prizes. Text stop 2 end.

Hookup: 1st a little about u. Text back GUY or GIRL then ur age. Like ‘GIRL 16’ or 'GUY 20'. Msg&rates apply.

Hookup: We want to know if we’re reaching youth all over CA. Text back ZIP then ur zipcode to tell us where ur at! Like ‘ZIP 94117’. Msg&rates apply

Hookup: How’d u get hooked up with the Hookup? A friend tell u? Teacher? Saw a poster? Text back HOOKED and ur reason, like ‘HOOKED my bff’. Msg&rates apply

Hookup: How has the Hookup changed u? Text back CHANGE then ur change or something new, like ‘CHANGE STD test last week’. Msg&rates apply.

Hookup: Thx 4 helping us make Hookup better 4u! Stay tuned 2 learn if u won our raffle 2 win $25 iTunes card. Back 2 ur regular tips next week! Msg&rates apply.

## Results

### Feasibility of SMS Evaluation

In total, 2477 Hookup subscribers were invited to participate in the survey, of those 832 (33.6%) responded and 58% (482) completed the full survey (responded to all four questions). Of all responses received, 85% were received within two hours,13% were received within the subsequent 22 hours, for a total of 98% of responses received within 24 hours.

Respondents were lost with each subsequent question, 22% were lost at second question, 34% at third and 50% at fourth question (cumulative).

### Evaluation of The Hookup

#### Question 1: Are We Reaching Target Population (california Youth)?

Analysis of the responses showed that of the people who responded, over 90% were in the age range of 14-21 with 92% girls and 8% boys. Of the people who responded, 95% lived in zip codes that were California-based. The results appear to indicate that The Hookup is reaching the target population of California youth with a higher female than male representation. This finding has guided improved program promotion to reach males.

#### Question 2: Are Our Promotion Efforts Successful?

Due to the multi-faceted promotion plan, the question assessing how users heard about the Hookup was left open-ended. The responses reflected the program’s promotion efforts. The main sources of referral to the campaign were: teachers/school (40%), friend (25%), posters/stickers/tattoos (22%), and online/web (10%). This has resulted in enhancing school-based partnerships for The Hookup promotion, as well as increased web-based promotion efforts.

#### Question 3: Are Our Messages Effective?

Of the respondents who answered the question about behavior change (n=482), 90% indicated that they had made some behavior change since they started receiving the text messages, including using condoms (33%), increased knowledge and awareness (24%), initiation of birth control (15%), and getting tested for HIV and other STDs (15%).

## Discussion

### Principal Results

Feasibility of SMS Evaluation: with traditional data collection methods facing modern day challenges, using text messaging for short surveys can be an effective and efficient method. This study demonstrates the feasibility of using text messaging for evaluating health campaigns in terms of response rates, turnaround time, and ability to reach the target audience. As compared to online surveys, the response rate was comparable and the turnaround time was much faster [12,13].

Evaluation of The Hookup: the data collected correlates with key Hookup program objectives, raising awareness/knowledge, increasing safer sex and condom use, and getting tested for STD/HIV among youth in California. The findings on sources of referral to Hookup indicate that teachers and other school staff have been key in promoting the uptake of this program among youth.

### Limitations

The challenges to using SMS as a survey tool include fitting questions and potential responses within the 160 character limit of text messaging and accounting for the loss of respondents as the number of questions increase. Our findings indicate the need to keep the surveys short and the questions clear to minimize drop-out rates. Additionally, the methodology may have presented a selection bias in those who chose to respond, potentially posing a threat to validity. Although preliminary calculations indicate that this method could be cost-effective, more in-depth study is needed to conduct a cost-effective analysis of text messaging for program evaluation.

### Conclusions

Using SMS messaging for prevention has high potential for reaching young people consistently and supporting their decision making process around their sexual and reproductive health. SMS messaging shows immense potential to increase access to free and low cost health care, and increase knowledge in the United States among hard to reach populations such as adolescents. With text messaging being seen as a low cost, viable method of reaching communities with valuable information and critical resources, this study sheds some light on actual field experience through a functioning statewide campaign, as opposed to a pilot demonstration project. Additionally, SMS is an innovative and effective tool for conducting evaluations of SMS-based programs.

## Abbreviations

CFHC: California Family Health Council
SMS: short message service
STD: sexually transmitted disease
YTH: youth+tech+health
